# ACTivity as medicine In Oncology for Head and Neck (ACTIOHN): Protocol for a feasibility study investigating a patient-centred approach to exercise for people with head and neck cancer

**DOI:** 10.1371/journal.pone.0289911

**Published:** 2023-08-25

**Authors:** Adrian W. Midgley, Andrew R. Levy, Simon N. Rogers, Rachel C. Brooker, Valerie Bryant, Mary Gemma Cherry, Steven Lane, Michael M. Nugent, Ruth Price, Andrew G. Schache, Bridget Young, Joanne M. Patterson

**Affiliations:** 1 Department of Sport and Physical Activity, Edge Hill University, Ormskirk, United Kingdom; 2 Health Research Institute, Edge Hill University, Ormskirk, Lancashire, United Kingdom; 3 Department of Psychology, Edge Hill University, Ormskirk, United Kingdom; 4 Arrowe Park Hospital, Wirral University Teaching Hospital NHS Foundation Trust, Liverpool, United Kingdom; 5 Faculty of Health and Social Care, Edge Hill University, Ormskirk, United Kingdom; 6 The Clatterbridge Cancer Centre NHS Foundation Trust, Wirral, United Kingdom; 7 Cancer of Head and Neck Group Experience (CHANGE) Patient and Public Involvement Group, Sunderland Royal Hospital, Sunderland, United Kingdom; 8 Institute of Population Health, University of Liverpool, Liverpool, United Kingdom; 9 Institute of Infection, Veterinary, and Ecological Sciences, University of Liverpool, Liverpool, United Kingdom; 10 Oral and Maxillofacial Surgery, Sunderland Royal Hospital, South Tyneside and Sunderland NHS Foundation Trust, Sunderland, United Kingdom; 11 Liverpool Head and Neck Centre, Liverpool University Hospitals NHS Foundation Trust, Liverpool, United Kingdom; 12 Department of Molecular and Clinical Cancer Medicine, Liverpool Head and Neck Centre, University of Liverpool, Liverpool, United Kingdom; 13 Liverpool Head and Neck Centre, School of Health Science, University of Liverpool, Liverpool, United Kingdom; University of Study of Bari Aldo Moro, ITALY

## Abstract

**Background and aim:**

Attempts at personalisation of exercise programmes in head and neck cancer (HaNC) have been limited. The main aim of the present study is to investigate the feasibility and acceptability of introducing a remotely delivered, fully personalised, collaborative, and flexible approach to prescribing and delivering exercise programmes into the HaNC usual care pathway.

**Methods:**

This is a single arm, feasibility study. Seventy patients diagnosed with HaNC will be recruited from two regional HaNC centres in the United Kingdom. Patients will undertake an 8-week exercise programme designed and delivered by cancer exercise specialists. The exercise programme will start any time between the time of diagnosis and up to 8 weeks after completing treatment, depending on patient preference. The content of the exercise programme will be primarily based on patient needs, preferences, and goals, but guided by current physical activity guidelines for people with cancer. The primary outcome measure is retention to the study. Secondary quantitative outcomes are uptake to the exercise programme, different measures of exercise adherence, pre- and post-intervention assessments of fatigue (Multidimensional Fatigue Symptom Inventory—Short Form), quality of life (SF-36), physical activity levels (International Physical Activity Questionnaire–Short Form), and various components of physical fitness. The outcomes of the nested qualitative study are acceptability and feasibility of the intervention evaluated via interviews with patients, health care professionals, and the cancer exercise specialists. Intervention and participant fidelity will be determined using checklists and scrutiny of each patient’s logbook and the cancer exercise specialists’ meeting notes. Analysis of quantitative data will be via standard summary statistics. Qualitative data will be analysed using thematic analysis.

**Expected results:**

This feasibility study will inform the design and conduct of a future randomised controlled trial. Success will be defined according to a traffic light system for identifying the appropriateness of progression to a randomised controlled trial.

**Trial registration:**

International Standard Randomised Controlled Trial Number registry (ISRCTN82505455).

## Introduction

### Rationale and background

Head and neck cancer (HaNC) represents malignancies arising from the larynx, hypopharynx, oropharynx, oral cavity, lips, and nasopharynx [1]. Globally, HaNC is the seventh most common cancer with over 930,000 new cases and almost 500,000 deaths annually [2]. Symptom burden is typically considerable and mainly related to the location of the cancer and its treatment, the latter of which is often aggressive and multi-modal [3]. Some symptoms are often still evident at 1-year post-treatment [4]. Notably, people with HaNC are typically frailer and more than twice as likely to report cancer-related disability than individuals with other cancers [5,6]. Identification of safe and effective interventions to help address these issues and improve the quality of life of people with HaNC is therefore an important endeavour.

Substantial scientific evidence accumulated for more than 30 years indicates that exercise is a safe, cost-efficient, and effective intervention during all parts of the cancer continuum [7]. Regular exercise can reduce symptoms such as cancer-related fatigue and depression, attenuate cancer treatment-related toxicity, help prevent and manage co-morbidities, and reduce cancer-specific and all-cause mortality [8–11]. Exercise oncology research has mostly been conducted in breast, prostate, and colon cancer, however, and HaNC has been severely underrepresented [12]. Generalising findings from other cancers is problematic, since people with HaNC often differ considerably in ways that have important implications for exercise prescription and delivery [13]. A survey of 430 people with HaNC, for example, found that HaNC-related symptoms were perceived as major barriers to engaging in exercise [14]. Specifically, dry mouth and throat, swallowing difficulties, shoulder weakness and pain, and drainage issues in the mouth or throat were ranked first, fifth, sixth, and seventh, respectively, of 37 perceived barriers to exercise. Other symptoms such as pain, depression, malnutrition, and weight loss are common to other cancers, but often more prevalent or more severe in people with HaNC [13]. This considerable symptom burden, combined with poor health literacy, high levels of social deprivation, and social isolation [15–17], likely explains the low levels of physical activity and poor cardiorespiratory fitness observed in HaNC cohorts [18,19]. Moreover, these factors make people with HaNC a hard-to-reach group for engagement in lifestyle interventions such as exercise programmes [16,20].

A survey of people with HaNC in the United Kingdom reported that many are motivated to participate in an exercise programme if such an intervention was available [14]. Physical activity is not included in the National Institute of Health and Care Excellence guidelines for improving HaNC outcomes [21], however, and exercise programmes are not currently part of usual care for HaNC in the United Kingdom. Moreover, despite their many unique issues that have important implications for exercise [13], no published physical activity guidelines currently exist on how to best prescribe exercise and promote its uptake and adherence in people with HaNC. Another notable issue is that centralisation of HaNC services in the United Kingdom has resulted in regional units serving large geographical areas. Thus, centralised service-based exercise programmes are unlikely to be an effective approach to support exercise uptake and adherence, since travel distance negatively impacts participation in an exercise programme in those with cancer [22].

A major criticism of exercise oncology research is that the individual needs of people with cancer have largely been ignored, using a ‘one size fits all’ approach to the design and delivery of exercise programmes [23,24]. Consequently, a patient-centred approach that includes personalisation of exercise programmes for people with HaNC has been limited [25–27]. This is an important issue, as preferences for the frequency, intensity, duration, type, and location of exercise, and timing of the start of an exercise programme, vary considerably among people with HaNC [14]. Studies involving mainly people with breast cancer have shown significantly better outcomes for personalised exercise programmes versus a more standardised approach [28,29]. A collaborative approach in which people with HaNC have at least equal input into the design of the exercise programme is also desirable, as this provides a high level of autonomy, which is an important determinant of exercise adherence [30]. This is consistent with the National Health Service’s long-term plan for a high level of personalisation in health and care in England, recognising that a one size fits all approach cannot meet the increasing complexity of peoples’ needs and expectations [31]. Another issue is that fluctuations in symptoms across the HaNC continuum affect the willingness, tolerance, and ability to perform exercise [32,33], which might be somewhat dependent on the type of treatment the patient is receiving or has received. It therefore seems desirable to incorporate built-in flexibility into exercise programmes, to allow patients to autoregulate their exercise in quick response to changing symptoms [34]. Built-in flexibility also should improve exercise adherence by allowing a rapid response to any changes in patient circumstances and by improving self-efficacy for overcoming barriers to exercise [35].

### Study aims and objectives

A fully personalised, collaborative, and flexible approach to exercise prescription in people with HaNC has not been investigated. Thus, the main aim of the present study is to investigate the feasibility and acceptability of introducing a remotely delivered, fully personalised, collaborative, and flexible approach to prescribing and delivering exercise programmes into the HaNC usual care pathway. This study will inform a future randomised controlled trial (RCT). Objectives are to determine:

1. Eligibility, exercise programme uptake, retention, and different aspects of exercise adherence.
2. People with HaNC, health professionals’, and cancer exercise specialists’ views on acceptability, intervention components, processes, and feasibility of integrating the intervention into the usual care pathway.
3. Intervention and participant fidelity.
4. Frequency, intensity, time, type, and location of exercise, and timing of the start of the exercise programme, based on the current needs, preferences, and goals of people with HaNC.
5. Suitability of outcome measures and provide data to allow a sample size calculation for a definitive RCT.

## Materials and methods

### Research design and study setting

**Fig 1:**
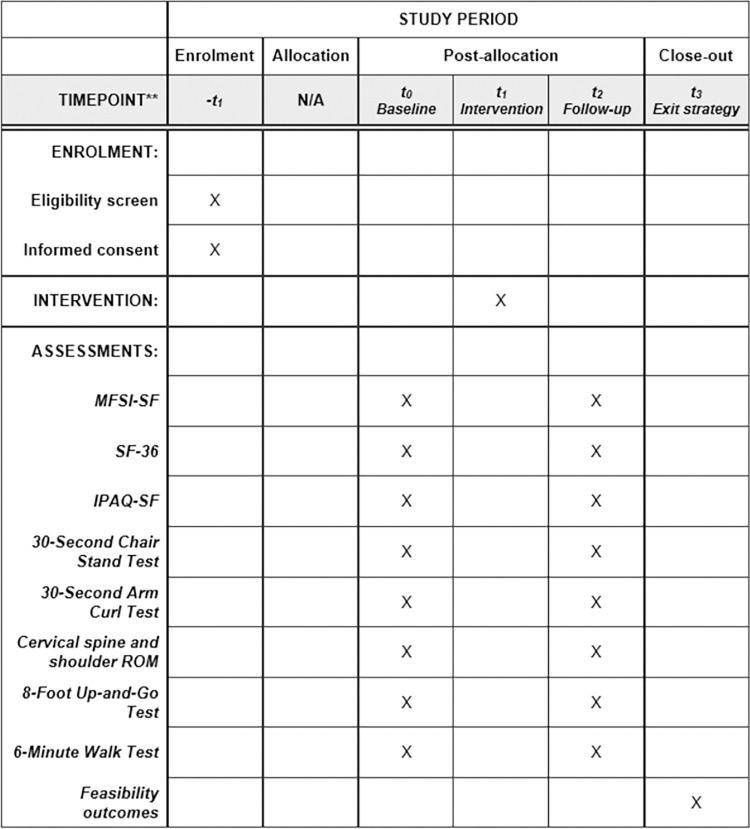
Spirit study timeline and data collection time points. N/A: Not applicable; MFSI-SF: Multidimensional Fatigue Symptom Inventory Short Form; SF-36: Short-Form 36 Health Survey Questionnaire; IPAQ-SF: International Physical Activity Questionnaire Short Form; ROM: range of motion.

**Fig 2:**
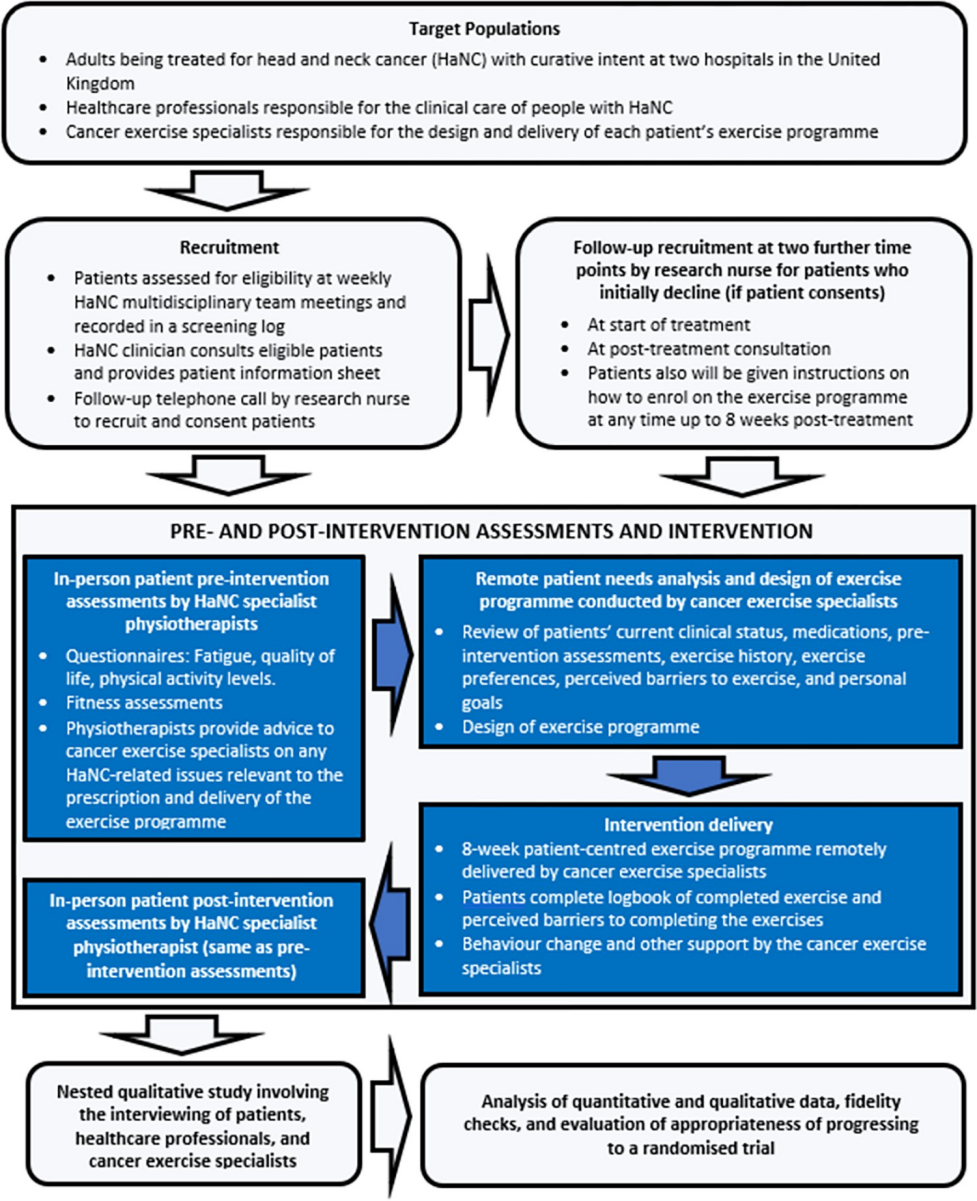
Flow diagram showing an overview of the study design and procedures.

This one arm, two-centre feasibility study, registered on the International Standard Randomised Controlled Trial Number registry (trial registration number ISRCTN82505455), will take place at two regional National Health Service HaNC centres. Aintree University Hospital, which is part of Liverpool University Hospitals NHS Foundation Trust, is located in Liverpool in the North West of England and is host to the largest single centre HaNC unit in the United Kingdom and in a geographical location that has the third highest multiple index of deprivation in England [36]. Sunderland Royal Hospital, which is part of the South Tyneside and Sunderland NHS Foundation Trust, is located in the North East of England. The Head and Neck Multidisciplinary Team serves the population of Sunderland, South Tyneside, and North Durham. Sunderland is one of the 20% most deprived districts/unitary authorities in England. Life expectancy for both men and women are lower than the average for England. Fig 1 shows the SPIRIT study timeline and data collection time points. An overview of the study design and procedures is shown in Fig 2.

### Participants

Patients. Seventy patients will be recruited from the two HaNC regional centres. This should provide a minimum sample size of 42 patient participants on study completion, based on a conservative estimated retention rate of 60% [15,18,26,37]. The target sample size was determined pragmatically using guidelines for feasibility studies that recommend a sample size of between 24 and 50 [38–40]. Moreover, a sample size of 40 people with HaNC for a feasibility study involving an exercise intervention has been deemed sufficient to estimate an effect size for a full trial [41].

Patient participants must meet the following inclusion criteria: 1) ≥ 16 years old; 2) able to provide informed consent; 3) diagnosed with HaNC and being treated with curative intent; 4) between the time of diagnosis and up to 8 weeks post-treatment; and 5) classified as low or medium risk according to a physical activity preparticipation risk stratification tool [42]. Patients classified as high risk according to the risk stratification tool, being treated with palliative intent, or engaging in any other intervention that may exert interference effects during their participation in this study, will be excluded from participation. The study will be as inclusive as possible, engaging adult patients with no restriction on age, socioeconomic background, ethnic diversity, or geographical location. The National Institute for Health and Care Research Equality, Diversity and Inclusion toolkit [43] will be used for increasing participation of underserved communities, such as ethnic minorities. Any patients with speech difficulties will be supported with alternative and augmentative communication methods.

Health care professionals and cancer exercise specialists. Up to 20 healthcare professionals, involved in the usual care of people with HaNC, will be consented to take part in an interview to establish their views on the acceptability, processes, and integration of the intervention into the usual care pathway. Three cancer exercise specialists, employed to design and deliver the exercise programmes, also will be invited to be interviewed.

### Recruitment

A HaNC research nurse or the treating clinician will screen potentially eligible patient participants against the eligibility criteria using weekly head and neck multidisciplinary team meetings, clinical lists, and patient medical notes. Eligible patients, who represent those newly diagnosed with HaNC, will be introduced to the study, and given participant information sheets for both the main study and the nested qualitative study during their next routine clinic appointment, between the time of diagnosis and the start of treatment. Eligible patients will be subsequently followed-up to enquire about their willingness to enrol. Although it has been recommended that exercise programmes should be included in the cancer survivorship plan as early as possible [44], many people with HaNC do not feel able to start before treatment [14]. Patients that initially decline will therefore be asked for consent to be re-approached after starting their cancer treatment and, where relevant, again at their first post-treatment consultation. Patients also will be informed that they can enrol in the exercise programme at any time between diagnosis and up to 8-weeks post-treatment and will be given instructions on how to do this. Patients will not be permitted to re-enrol in the exercise programme if they withdraw, or postpone the exercise programme once started, since the effect of timing of the start of the exercise programme on exercise adherence will be evaluated. Allowing different starting time points also allows for identification of any issues that are specific to the starting time. Patients that decline the intervention, or who withdraw from or postpone the intervention, will still be offered the opportunity to consent to participate in the nested qualitative study.

The rate and timing of enrolment onto the exercise intervention will be quantified. Reasons for refusal to enrol onto the exercise programme will be sought while respecting the right to refuse without giving a reason. Patients who provide written consent to participate will be referred to an oncology specialist physiotherapist for pre-intervention assessments and their general practitioner will be informed about their participation in the study. Patients are not expected to incur any personal expenses during any stage of the study and there will be no financial compensation for participation. The study is suitably insured against personal harm.

### Ethics

Favourable ethical opinion has been given by the West of Scotland Research Ethic Service (reference 22/WS/0058). Any protocol modifications will be approved by this committee and the sponsor will be notified. All participants will agree to participate by signing a statement of informed consent. Patients will provide separate consent for participation in the intervention and interview.

### Pre- and post-intervention assessments

Assessments will be conducted in clinic by oncology specialist physiotherapists immediately before and after the 8-week exercise programme and will consist of patient-reported outcome measures and objective measures (Table 1). After baseline assessments, patients will be referred to a cancer exercise specialist for a needs analysis and subsequent design and delivery of the exercise programmes.

**Table 1:** Measurement properties of the pre- and post-intervention assessments. Assessments of physical fitness will be performed in the order shown in the table in accordance with recommendations [45].

Outcome	Measurement Tool	Attributes
Fatigue	Multidimensional Fatigue Symptom Inventory—Short Form (MFSI-SF)	Comprises 30 statements or questions with a 5-point Likert-scale scoring response that assesses fatigue across five domains: 1) general fatigue; 2) physical fatigue; 3) emotional fatigue; 4) mental fatigue; and 5) vigour [46].
Health-related quality of life	Short-Form 36 Health Survey Questionnaire (SF36)	Comprises 36 questions that explore eight domains of health: 1) physical functioning; 2) role limitations due to physical health; 3) role limitations due to emotional problems; 4) energy/fatigue; 5) emotional well-being; 6) social functioning; 7) pain; and 8) general health [47].
Physical activity levels	International Physical Activity Questionnaire (IPAQ)–Short Form	Comprises seven questions that explore the amount of moderate and vigorous physical activity performed and the amount of time spent walking and sitting over the past 7 days [48].
Lower body muscular strength and endurance	30-Second Chair Stand Test	Maximum number of times within 30 s that an individual can rise from a seated position to a full stand without pushing off with the arms [45]. ICC = 0.89 [49].
Upper body muscular strength and endurance	30-Second Arm Curl Test	Maximum number of times a hand weight can be curled through a full range of motion in 30 s. Hand weight is 5 lb for women and 8 lb for men [45]. ICC = 0.81 [49].
Cervical spine and shoulder range of motion	Long-arm goniometer	Cervical spine range of motion in forward flexion, extension, lateral flexion, and rotation. Shoulder range of motion in flexion, abduction, and lateral and medial rotation [50].
Power, speed, agility, and dynamic balance	8-Foot Up-and-Go Test	Shortest time taken to rise from a seated position on a chair, walk 8 feet around a cone, and return to the chair and sit back down [45]. ICC = 0.95 [49].
Aerobic endurance	6-Minute Walk Test	Maximum distance that can be walked in 6 minutes along a 30 m course [51]. ICC = 0.94 [49].

**ICC** = test-retest intraclass correlation coefficient.

### Study intervention

The intervention is a personalised, collaborative, and flexible 8-week exercise programme, delivered remotely to address the issue of regional cancer units serving large geographical areas. Each exercise programme will be co-designed by the patient and a cancer exercise specialist and informed by a comprehensive individual needs analyses that considers each patient’s current clinical status, medications, pre-intervention assessments, exercise history, exercise preferences, perceived barriers to exercise, and personal goals. The physiotherapists will provide advice to cancer exercise specialists on any HaNC-related issues identified in the pre-intervention assessments.

Exercise programmes will be developed within the framework of current physical activity guidelines for people with cancer [34,52,53], which state they should engage in aerobic, resistance, and flexibility training [34,52,53]. This is summarised in Table 2 according to the FITT method.

**Table 2:** Framework used in the current study for prescribing exercise according to the FITT (frequency, intensity, time, and type) method. How appropriate patterns and progressions of exercise are to be identified are also included.

Aerobic exercise	**Frequency:** ≥ 3 days/week. **Intensity**: Moderate intensity. Where moderate intensity exercise is not feasible/appropriate due to deconditioning or current health conditions, prescribe light intensity exercise. **Time:** ≥ 30 min duration, although deconditioned/currently sedentary individuals will likely need to start with less than 30 min. Between 10 and 20 min is an appropriate starting point for these individuals. **Type:** Exercise involving large muscle groups. **Pattern:** Avoid two consecutive days with no exercise. For deconditioned/currently sedentary individuals, consider intermittent exercise to accumulate the daily prescribed exercise time. **Progression:** Should involve a maximum 5 min increase in session duration each week, as tolerated, until 30 min of continuous exercise can be performed [54]. Exercise intensity should then be increased using the exercise intensity domains for ratings of perceived exertion proposed by the American College of Sports Medicine: light = 9–11; moderate = 12–13; vigorous = 14–17 [54].
Resistance exercise	**Frequency**: ≥ 2 days/week. Advise twice per week where possible. If a participant refuses to perform aerobic exercise, advise 3 days/week. **Intensity**: 8–15 repetitions (≥ 60% 1RM). Advise 10–15 repetitions for deconditioned/currently sedentary individuals. **Time:** For deconditioned/currently sedentary individuals, prescribe 1 set per exercise for the first 2 weeks and progress to 2 sets thereafter. Rest 2–3 minutes between sets. **Type**: Dynamic exercises using both concentric and eccentric muscle actions, although a combination of dynamic and isometric exercise can be performed if isometric exercise is not contraindicated. All major muscles groups should be exercised, preferably using multijoint exercises. **Pattern:** Spread sessions evenly throughout the week, with at least 48 hr recovery before exercising the same muscle groups again. **Progression:** Use the double progressive method [55]. This initially involves increasing the repetitions within the target repetition range. Once the patient can perform the maximum number of repetitions within the target repetition range for all sets for a given exercise, with good form, the resistance is increased. When using exercise bands, the patient should be instructed on the importance of standardising hand placement on the band. Hands that are further apart on the band will increase resistance, and vice-versa. **Miscellaneous:** The resistance training programme should include 8–10 different exercises using a whole-body approach. Split routines should be avoided unless the participant has recent (within the last 4 weeks) resistance training experience, and it is inappropriate for the volume of resistance exercise to be fit into one training bout. Exercises that target specific muscles should be incorporated into the training programme where dysfunction from treatment is interfering with the ability to undertake activities of daily living. Multijoint exercises and large muscle groups should be exercised before single joint and smaller muscle groups in each exercise session.
Flexibility exercise	**Frequency:** ≥ 2 days/week on days when other exercise is being performed. **Intensity:** Stretch to a feeling of tightness or slight discomfort. **Time:** 8–10 stretches each held for 10–15 sec for maintenance stretches and 30 sec for developmental stretches. **Type:** Static stretches for major muscle groups, focusing more time on any muscles that have been found to be shorter than the normal range, especially if causing physical dysfunction.

Balance exercises also will be encouraged for any patients that present with balance issues during their initial assessments [53]. Patients’ needs, preferences, and goals will take precedence, however, if these are not congruent with current physical activity guidelines. This approach is based on the premise that the best exercises are the ones the person will do. Personalisation of exercise programmes will include appropriate manipulation of the frequency, intensity, time, type, volume, progression, and location of the exercise [54]. A menu of no-cost, convenient, locally available, or virtual exercise options, partly informed by previous research [14], will be used to help patients choose their preferred type of exercise and location. However, patients will be encouraged to suggest alternatives based on preferences and any past successes at adhering to exercise. The exercise location is therefore likely to differ based on patient preference. Whether patients exercise alone or with others also will largely depend on personal preference. Supervised group exercise sessions will not be offered due to increased expense and funding restrictions. Flexibility will be embedded into each patient’s exercise programme so that they can autoregulate the acute training variables in response to changes in symptoms and other perceived barriers to exercise [34]. Coloured elastic resistance bands (Theraband, Akron, OH) will be given to patients for performing resistance exercises. Each colour represents a different band resistance at 100% elongation (i.e., tan = 1.1 kg, yellow = 1.3 kg, red = 1.7 kg, green = 2.1 kg, purple = 2.6 kg, black = 3.3 kg, silver = 4.6 kg, and gold = 6.5 kg). Resistance bands have been shown to be a versatile and effective method for developing muscular strength and endurance compared to the use of conventional resistance training equipment such as free weights and resistance training machines [56].

Exercise programmes will be progressed towards meeting the minimum recommended amount of physical activity for people with cancer [52] where appropriate. The focus, however, will be on avoiding inactivity and promoting exercise adherence rather than meeting the physical activity guidelines as a primary goal. Exercise programmes will be progressed by manipulating only one acute training variable at a time.

Comprehensive instructions will be given to patients on how to interpret the 6–20 ratings of perceived exertion scale, according to those published by Borg [57]. An ‘ACTIOHN exercise prescription principles’ document, written specifically for this study, will be used to guide the cancer exercise specialists when writing each patient’s exercise programme and for completing fidelity checks.

Each patient will meet with a cancer exercise specialist before the exercise programme starts, to discuss the exercise programme and to obtain patient approval of its content, and for the cancer exercise specialist to provide instruction on exercise technique and provide information on safety. During the 8-wk exercise programme, a cancer exercise specialist will undertake a weekly 30-minute, individual video or telephone consultation with each patient. Consultations will provide patient education, discuss safety issues, address perceived exercise barriers, and encourage accurate and timely completion of the patient’s daily exercise log. Videos, delivered via a commercially available app (Physitrack®; Physitrack PLC, UK), will be used to help instruct patients on how to perform exercises safely and effectively. Paper copies showing photographs and written instructions for each exercise will be supplied to any patients where video access is not possible. Although all exercises will be performed by patients unsupervised, the cancer exercise specialists will check and advise on technique during weekly consultations. The cancer exercise specialists will send two texts each week to each patient to further encourage timely completion of daily exercise logs. The second text also will include a motivational message.

Behaviour change techniques to improve the patients adherence to the exercise programme will draw on the CALO-RE taxonomy [58] and will be implemented using motivational interviewing skills [59]. Behaviour change techniques and motivational interviewing will be informed by Self-Determination Theory [60]. The cancer exercise specialists have been given specialist training on behaviour change techniques relevant to physical activity and their implementation through the motivational interviewing approach. Further behaviour change support for patients will include being given written ‘top tips’ information and ‘exercise stories’ of how to best manage specific HaNC symptoms and overcome barriers to exercise. These peer-informed resources were developed by clinicians in collaboration with the Cancer of Head and Neck Group Experience (CHANGE) patient and public involvement (PPI) group. The CancerFit (www.cancerfit.me/site/) and Macmillan (www.macmillan.org.uk/cancer-information-and-support) websites, and our Twitter feed will provide online resource support.

After post-intervention assessments with the oncology specialist physiotherapist, the patient will meet with a cancer exercise specialist for a final time to develop and agree a personalised exit strategy from the 8-week exercise programme. This is to facilitate transition to independent long-term exercise and will involve discussing and formalising the patient’s goals over the next 12 months, how the current exercise programme can be progressed, and information to facilitate sustained behaviour change and how to deal with relapses. This use of an exit strategy is consistent with that recommended for exercise referral schemes in the United Kingdom [61].

### Support for the cancer exercise specialists

A certified cancer exercise specialist is an advanced fitness instructor with a level 4 vocational qualification, according to the Regulated Qualifications Framework in the UK, in designing and delivering oncology exercise programmes. The three cancer exercise specialists involved in the present study also hold other vocational health and fitness qualifications and one is enrolled on an MSc in Cancer Care degree programme. All the cancer exercise specialists performed a similar role as for the present study working on the SafeFit trial [62]. They already have received training from a chartered psychologist (ARL) in motivational interviewing and other behaviour change techniques. The training involved two, 2-hour workshops and included a bespoke workbook that contains content from workshop presentation slides, practical activities, scenario-based learning activities, action and coping planning sheets, and self-reflective diary sheets. The cancer exercise specialists also met with the chartered psychologist for 1 hour at another two time points during working with their first patient to obtain further support in applying the motivational interviewing and other behaviour change techniques. Exercise programme design and delivery by the cancer exercise specialists will be undertaken with the guidance and supervision (including fidelity checks) of a certified exercise physiologist (AWM) via fortnightly meetings throughout the study period.

### Outcome measures

Primary quantitative outcome. Retention: Percentage of patients that completed the 8-week exercise programme.

### Secondary quantitative outcomes

1. Exercise uptake: percentage of eligible patients that agreed to participate in the exercise programme. Reasons for refusal will be documented where possible.
2. Exercise adherence (based on the definitions proposed by Nilsen et al. [63]):
  - Exercise programme adherence: percentage of the total prescribed training sessions engaged in.
  - Exercise programme interruption: percentage of patients that missed at least three continuous scheduled training sessions.
  - Pre-session dose modification: number of training sessions where the exercise dose was modified before starting the training session.
  - During session dose modification: number of training sessions where the exercise dose was modified during the training session.
  - Early session termination: number of training sessions terminated early by the patients.
3. Pre- and post-intervention patient-reported measures of fatigue (MFSI-SF), quality of life (SF-36), and physical activity levels (IPAQ-SF).
4. Pre- and post-intervention measures of physical fitness: aerobic endurance (6-Minute Walk Test), upper and lower body muscular strength and endurance (Arm Curl Test and 30-Second Chair Stand Test), power, speed, agility, and dynamic balance (8-Foot Up-and-Go Test), and flexibility (goniometric measures of neck and shoulder range of motion).
5. Frequency, intensity, duration, type, and location, of exercise, and the starting point of the exercise programme (i.e., before, during, or after treatment).

Qualitative outcomes. Acceptability, processes, and integration of the intervention into the usual care pathway will be evaluated by interviewing a sample of patients, health care professionals, and the cancer exercise specialists.

### Data collection methods

The two HaNC centres will keep logs on the number of eligible patients, and numbers screened, approached, and consented. Reasons for ineligibility will be recorded where relevant. Patient data will be recorded on case report forms, which will be checked for completeness and legibility before being entered into a trial database. Database entries will be checked for accuracy against the case report forms for all primary outcomes and 20% for secondary outcomes. If there are database errors in the primary outcomes, or greater than 1% errors for secondary outcomes, 100% data checks will be completed until the error rate ceases or drops.

Physical fitness tests will be standardised using a document that describes 1) the information to be given to patients at least 48 hours before arriving at the laboratory to prepare for the tests; 2) general safety precautions; 3) details of the standardised pre-test warm-up; and 4) how each test should be conducted, including the mandatory test order. The fitness test procedures are based on those described by Rikli and Jones [45] and Norkin and White [50]. Each patient will record the exercise intensity, duration, sets, and repetitions (as appropriate) of every exercise session in a logbook during the 8-week exercise programme. Patients also will record any adverse symptoms, other issues, and reasons for non-adherence. Patient demographic and characteristic data will be collected from patient medical records and include age, sex, ethnicity, history of smoking and alcohol consumption, tumour site and stage, human papillomavirus status, cancer treatment type, comorbidities, and body mass index. Socioeconomic status will also be determined using the participant’s full home postcode and reported as the English Index of Multiple Deprivation score.

A summary of the data collection methods to address each of the study’s objectives is shown in Table 3.

**Table 3:** Methods for collecting data for each study objective.

Study objective	Method of recording
Eligibility, exercise programme uptake, retention, and different aspects of exercise adherence.	Screening logs, patient participant tracking log, and patient participant logbooks.
People with HaNC, health professionals’, and cancer exercise specialists’ views on acceptability, intervention components, processes, and feasibility of integrating the intervention into the usual care pathway.	Qualitative analysis of semi-structured interviews.
Intervention and participant fidelity.	Intervention fidelity: Completion of a checklist for each patient participant used to scrutinise exercise programmes and logs completed by the cancer exercise specialists. Another checklist will be used to document the training and support received by the cancer exercise specialists. Participant fidelity: Scrutiny of the participants’ logbooks after exercise programme completion.
Frequency, intensity, duration, type, and location of exercise, and timing of the start of the exercise programme, based on the current needs, preferences, and goals of people with HaNC.	Self-reported by patient participants in their exercise programme logbook.
Suitability of outcome measures and provide data to allow a sample size calculation for a definitive RCT.	Quantitative data and qualitative analysis of semi-structured interviews.

**HaNC** = head and neck cancer.

### Fidelity

Intervention fidelity will be determined using a checklist for each patient. Completion of this checklist will be facilitated by scrutinising exercise programmes and logs completed by the cancer exercise specialists. The logs will include meetings notes and records of texts sent to each participant. Another intervention fidelity checklist will be used to document the training and support received by the cancer exercise specialists. Participant fidelity will be determined by scrutiny of the participants’ logbooks after exercise programme completion.

### Interviews

Acceptability of the intervention will be explored by qualitative semi-structured interviews with approximately 20 of the participants with HaNC, purposively sampled to ensure maximum variation based on gender, age, socio-demographic characteristics, location, type of cancer treatment, and where possible, those that declined to participate. Patients who decline, withdraw from, postpone, or complete the intervention will be invited to participate in an interview. Purposive sampling will aim to capture patients at different time points (i.e., both during and after completion of the programme) and patients with different levels of adherence to the intervention. Feasibility of integrating the intervention into the usual care pathway will be explored by qualitative semi-structured interviews with up to 20 healthcare professionals involved in the care of the people with HaNC at the two study sites, and the cancer exercise specialists. The focus will be on assessments and programme delivery, usability of the study materials and intervention tools, and acceptability of the content and timing of the intervention. All interviews will be conducted by an experienced qualitative researcher with skills in interviewing vulnerable populations around sensitive topics. Informed consent will be sought prior to interview. Interviews will be audio-recorded. A topic guide will be used in the interviews, but interviewees will be encouraged to speak freely about any other issues relating to the feasibility and acceptability of the intervention. The topic guide has been developed from discussions within the wider research team, the CHANGE PPI group, and from literature around exercise participation. The guide will be revised as new issues emerge in each interview. Each interview is expected to last between 40 and 60 minutes.

## Data analyses

Quantitative methods. The primary outcome is to report the retention rate of patients enrolled into the study. The retention rate will be estimated to a degree of precision equal to 11.5% if the minimum sample size is achieved. As this is a feasibility study, no formal hypothesis tests will be undertaken. Both primary and secondary outcomes will be reported for baseline and follow-up using standard summary statistics such as means, medians, and percentages, along with corresponding 95% confidence intervals. Demographic information also will be reported using standard summary statistics. Estimates of variability in the primary outcome measure will be used to determine the sample size for a definitive randomised controlled trial. All analyses will be undertaken using the STATA software package (Version 17, StataCorp LLC, Texas, USA).

Qualitative methods. Thematic analysis will be undertaken using a framework approach [64,65]. Data will be analysed by the Research Associate and team members (AL, AWM, VB, MGC, JP), and conducted iteratively throughout the project. The developing analysis will be regularly discussed at project meetings and where appropriate, inform changes to study processes. The patient and staff transcripts will initially be analysed separately. The enquiry will focus on the feasibility and acceptability of introducing exercise using a personalised, collaborative, and flexible approach, at a range of time points, including the exit strategy and exercise maintenance. The analysis team will discuss and agree emerging codes and themes. The analysis framework will then be constructed, informed by Normalization Process Theory [66], covering four key areas: 1) how people make sense of a new practice (coherence); 2) the willingness of people to sign-up and commit to the new practice (cognitive participation); 3) their ability to take on the work required of the practice (collective action); and 4) activity undertaken to monitor and review the practice (reflexive monitoring). The analysis will remain open to relevant issues that may not be captured within the framework and novel issues arising in transcripts incorporated into a revised framework. Standard approaches to the rigorous analysis of qualitative data (including constant comparison, deviant case analysis, and member checking for accuracy [67]) will be used.

Triangulation. Quantitative and qualitative data on patients’ and healthcare professionals’ engagement with the intervention, will be regularly discussed and triangulated at project management group meetings to identify ways to refine the intervention and study processes while the study is ongoing. The impact of these refinements will then be examined both qualitatively and quantitatively. Procedurally, the “following a thread” framework for triangulation and integrative analysis of qualitative and quantitative data will be adopted. There is no assumption over the primacy of either data type and will look for both convergences and divergences between them and aim to produce categories and themes that accommodate both types of data. Attention to exploring any divergences between the quantitative and qualitative data will be given, as these can often provide important insights. This will generate insights about ways of personalising the intervention to the needs and preferences of different patient groups, understanding variation in engagement and outcomes of the intervention, and provide insights on the mechanisms by which the intervention has an impact.

### Data management

Patients’ study data will be collected by the principal investigator for each study site, or their delegated representative, and recorded in the case report forms. The case report form data will be pseudo-anonymised through a unique study identifier number. A record linking the patient’s name to the unique study identifier number will be held in a locked room at the study sites and will be the responsibility of the principal investigators. The Chief Investigator or delegated representative will monitor completeness and quality of data recording in case report forms and will regularly correspond with site principal investigators (or delegated team members) with the aim of capturing any missing data and ensuring continuous high quality of data. Data will be entered at study sites onto a secure online system, with the paper originals remaining at site. Written consent forms will be stored securely according to the Research Ethics Committee’s requirement.

Interview audio recordings will be encrypted, and password protected and sent to the University of Liverpool for transcribing. Interview recordings will be transcribed verbatim by a professional transcription agency with a secure upload facility. Once received, transcripts will be checked for accuracy and edited to remove any identifying information to ensure anonymity of respondents. Each patient will be assigned a code that will give assurance that they cannot be identified. This will be stored with electronic consent records in case a participant wishes to withdraw from the study. Transcripts will be held securely on the password-protected encrypted server at the University of Liverpool. Recordings will be deleted once it is established that the research team will not need to revisit them.

The Chief Investigator will have overall responsibility for the quality and retention of study data. Data will be handled, computerised, and stored in accordance with the latest directive on Good Clinical Practice (2005/28/EC) and local policy. Clinical information will not be released without the written permission of the patient, except as necessary for monitoring and auditing by the Sponsor, its designee, regulatory authorities, or the Research Ethics Committee. Prior written agreement from the Sponsor or its designee must be obtained for the disclosure of any confidential information to other parties.

### Audit

The study may be subject to inspection and audit by the University of Liverpool under their remit as sponsor, and other regulatory bodies, to ensure adherence to Good Clinical Practice and the United Kingdom Policy Framework for Health and Social Care Research (v3.2., 10^th^ October 2017).

### Steering group

A Steering Group that includes external members not directly involved in the conduct of the study will oversee and advise on the study. They will meet quarterly to review findings, discuss interpretation, agree dissemination strategies, evaluate feasibility, and decide on the next phase of the project.

### Safety and adverse events

To minimise risks, patients will be evaluated using a physical activity preparticipation risk stratification tool [42], along with clinical judgement regarding suitability for enrolment onto the study via discussions at multidisciplinary team meetings. Each patient will be given a ‘Physiotherapy Advice and Information’ sheet that provides information on recognising signs and symptoms for which the patients should seek immediate medical attention during the intervention. The physiotherapists will be the main contact for reporting adverse symptoms; however, out-of-hours contact details also will be provided in the ‘Physiotherapy Advice and Information’ sheet. The cancer exercise specialists will be responsible for weekly monitoring of patients during their weekly meetings, including enquiring about any changes in health status, medications, and symptoms.

The principal investigator for each study site will be responsible for managing adverse events at site according to the protocol. Any serious adverse events that occur from baseline up until the last follow-up will be reported to the Chief Investigator and study sponsor within 24 hours and reported to the NHS Health Research Authority within 15 days of the Chief Investigator becoming aware of the event. If necessary, urgent safety measures will be implemented to protect patients against any immediate risk to their health and safety. The Health Research Authority will be notified immediately and, in any event within 3 days, if any such measures have been implemented and the reasons why.

### Role of study sponsor and funder

The study sponsor and funder have had no role in the design of the study or the writing of this protocol paper and will not be involved in the collection, management, analysis, and interpretation of the data, writing of subsequent papers, or the decision to submit papers for publication.

### Patient and public involvement

Details of PPI are shown in Table 4.

**Table 4:** Details of patient and public involvement (PPI) in the ACTIOHN study.

1: Aim	Input into identifying and prioritising research questions and then input into all stages of the research process.
2: Methods	VB took part in several early meetings with clinicians, exercise specialists, dieticians, etc, looking at possible priorities for exercise in head and neck cancer (HaNC). She also obtained feedback from questions she asked of patients at a survivorship day relating to their exercise experiences. The PPI group were then asked to provide input into identifying research questions relating to the role of exercise in HaNC. They also were asked for their opinions on completing specific fatigue and physical activity questionnaires. The group were further asked to develop resources that will be given to patient participants to advise them on how to best manage specific HaNC symptoms and overcome exercise barriers. This was overseen by clinicians. VB chaired three PPI meetings as part of the input into developing this study and corresponded with the group by email to obtain feedback outside of scheduled meetings. She attended the research team meetings and will continue to do so throughout the data collection, analyses, and dissemination phases. VB will chair another five PPI meetings during this period. She will link with the NIHR North West ARC Public Involvement and Engagement team to capitalise on their expertise and networks to ensure greater inclusivity and to ensure all voices are heard. VB will be involved in the qualitative data analysis and the PPI group will advise on dissemination plans.
3: Results	The PPI group stated that exercise is an important topic, as exercise was seen as a crucial part of facilitating a return to ‘normal life’. Some of the PPI group expressed uncertainties as to whether they could exercise, and some thought they needed to rest to recover. There were many exercise barriers described (e.g., confidence, motivation, leaving the house, feeding tube management, dry mouth, shoulder and neck stiffness, and finding achievable forms of exercise). Those that sought information about exercise in the past struggled to find anything appropriate for HaNC issues. They said a good assessment and encouragement from someone qualified, with suggestions for overcoming barriers to exercise (particularly relating to treatment side-effects), and an exercise programme flexible enough to accommodate their abilities, would be helpful. They were uncertain as to what the best time point might be to introduce an exercise programme. The PPI group piloted the patient-reported outcome measures and found them acceptable. Most of the PPI group preferred the short form version of the Multidimensional Fatigue Symptom Inventory over the Piper Fatigue Scale because it was easier to understand and quicker to complete. Most also preferred the short form version of the International Physical Activity Questionnaire over the long-form version because of the lower participant burden and thought this was particularly important when completing it when feeling fatigued. They also suggested the delivery of the intervention is acceptable and potentially achievable. The PPI group developed bespoke ‘top tips’ information and ‘exercise stories’ in collaboration with clinicians. These resources will be given to patient participants during their initial assessment visit to help them manage specific HaNC symptoms and overcome barriers to exercise.
4: Discussion	The PPI was effective in refining the research questions, study design, and study materials and procedures, and essential to the development of written resources to be given to patient participants, as detailed in section ‘3’. The views of the PPI group members were relatively consistent in regards helping identify appropriate research questions, materials, and procedures.
5: Reflections	Not applicable, as this is a protocol paper.

### Reporting guidelines

To facilitate clear reporting, this protocol paper used the following three checklists: Standard Protocol Items: Recommendations for Intervention Trials (SPIRIT) [68]; Consensus on Exercise Reporting Template (CERT) [69]; and the short form version of the Guidance for Reporting Involvement of Patients and the Public (GRIPP2-SF) [70].

### Status and timeline

Recruitment of patient participants began in October 2022 and is expected to be completed by October 2023. The data analysis is expected to be conducted by December 2023, and the manuscript(s) containing the quantitative and qualitative results submitted for publication in peer-reviewed journals in February 2024. Results will concurrently be made available to those involved in the care of people with HaNC and relevant patient forums.

### Success criteria

The success of this feasibility study will be defined according to the traffic light system proposed by Avery et al. [71] for identifying the appropriateness of progression to an RCT:

- **Green** (go—progression to an RCT with minimal changes to the protocol): Proposed sample size is achieved and retained, the intervention is considered acceptable and feasible by healthcare professionals and patient participants with only minor changes required, study procedures are considered acceptable and feasible, > 85% of outcome data are collected, an appropriate primary outcome measure has been selected, and ≥ 33% of those identified as eligible and approached were recruited.
- **Amber** (amend—progression to an RCT is appropriate but with minor changes to the protocol): At least 20 patient participants were recruited and retained at follow-up, moderate changes to the protocol and/or intervention are required, and > 60% of outcome data were collected.
- **Red** (stop—progression to an RCT inadvisable as major changes required): < 20 patient participants were recruited and retained at follow-up, significant changes to the protocol and/or intervention are required, and < 60% of outcome data were collected.

## Conclusion

To our knowledge, this will be the first study to investigate a fully personalised, collaborative, and flexible approach to prescribing and delivering exercise programmes for people with HaNC. The rationale for this patient-centred approach is that it should better meet the needs, preferences, and goals of the patients. A key aspect of the study is to determine the acceptability of the intervention and the feasibility of integrating it into the usual care pathway. This feasibility study will inform an RCT.
